# PIK3CA somatic alterations in invasive breast cancers: different spectrum from Caucasians to Chinese detected by next generation sequencing

**DOI:** 10.1007/s12282-020-01199-5

**Published:** 2021-01-01

**Authors:** Minghan Jia, Ning Liao, Bo Chen, Guochun Zhang, Yulei Wang, Xuerui Li, Li Cao, Hsiaopei Mok, Chongyang Ren, Kai Li, Cheukfai Li, Lingzhu Wen, Jiali Lin, Guangnan Wei, Charles M. Balch

**Affiliations:** 1 Department of Breast Cancer, Cancer Center, Guangdong Provincial People’s Hospital, Guangdong Academy of Medical Sciences, 106 Zhongshan Er Road, Guangzhou, 510080 Guangdong China; 2 School of Medicine, South China University of Technology, Guangzhou, China; 3 The Second School of Clinical Medicine, Southern Medical University, Guangzhou, China; 4 Department of Surgical Oncology, The University of Texas MD Anderson Cancer Center, Houston, TX USA

**Keywords:** PIK3CA, Somatic alteration, Sequencing, Breast cancer, TCGA

## Abstract

**Purpose:**

Somatic alteration of phosphatidylinositol-4,5-bisphosphate 3-kinase catalytic subunit alpha (PIK3CA) is a crucial therapeutic target in breast cancer (BC) and PI3Kα-specific inhibitor Alpelisib has been used in clinics. This study investigates the PIK3CA alterations in Chinese and Caucasians BC patients for the purpose of selecting anti-PI3K therapy.

**Methods:**

The molecular profile of the PIK3CA gene was analyzed in 412 Chinese patients with untreated invasive BC using a 540 gene next-generation sequencing panel. The results were compared with data of the Caucasian BC patients in The Cancer Genome Atlas (TCGA-white).

**Results:**

PIK3CA alterations were frequently found in BC of estrogen receptor (ER) positive (49.3%, *p* = 0.024), low ki67 proliferation index (58.3%, *p* = 0.007) and low pathological grade (grade I/II/III 80%, 53.4%, 35.9%, *p* < 0.001). Compared to TCGA-white, Chinese BC patients had a higher alteration frequency (45.6% vs. 34.7%, *p* < 0.001) with larger proportion of p.H1047R mutation among three common mutation sites (p.E545K, p.E542K and p.H1047R) (66.1% vs. 43.7%, *p* = 0.01). Across four molecular subtypes, ER + /human epidermal growth factor receptor 2 positive (HER2 +) tumors harbored the most PIK3CA alterations (51.6%), while ER-/HER2- harbored the least alteration (30.0%) but the most copy number amplification (19.05%).

**Conclusion:**

PIK3CA alterations prevail in Chinese BC patients and have different molecular features compared to that of Caucasians. The results provide precise annotations of PIK3CA genomic alterations of Chinese in the context of application of PIK3CA inhibitor.

## Introduction

Breast cancer (BC) is currently the most common cancer among women worldwide [1]. In addition to the harmful impact by industrialization induced pollutants or mental-related endocrine change, BC is well known as a complex and heterogeneous disease driven by genomic variants [2]. Statistically, 5–10% of breast cancers are primarily caused by genetic factors resulting from the accumulation of acquired somatic alterations [3].

Somatic alteration of the phosphatidylinositol-4,5-bisphosphate 3-kinase catalytic subunit alpha (PIK3CA) gene is one of the most prevalent driver genes in breast cancer, occurring at a frequency of 20–40% [4, 5]. PIK3CA gene is located in chromosome 3q26.32 and encodes the PIK3CA, also named the p110α protein, which is an important component of class I phosphoinositide 3-kinase (PI3K). More than 80% of PIK3CA mutations occur at three ‘‘hotspots’’, E542K and E545K in exon 9 encoding the helical domains, and H1047R in exon 20 encoding the kinase domains [6]. Through the PI3K/AKT pathway, altered PIK3CA gene plays a critical role in cell survival, apoptosis, proliferation, motility, and adhesion [7].

PI3K inhibition as a therapeutic strategy for BC has been investigated throughout many clinical trials [8, 9]. Theα-specific PI3K inhibitor Alpelisib (PIQRAY, Novartis) has received accelerated approval by the Food and Drug Administration (FDA) in May 2019 for the treatment of patients with estrogen receptor–positive (HR +)/human epidermal growth factor receptor 2-negative (HER2-) PIK3CA-mutated advanced or metastatic BC based on the SOLAR-1 trial [10]. With the development of next-generation sequencing (NGS) technologies, more recognition on the individual genomic landscape has become possible. Some studies have focused on the altered PIK3CA gene spectrum in different race [11, 12]. However, there is a lack of comprehensive comparison of the PIK3CA gene alteration spectrum between Caucasians and Chinese in a large scale.

In this study, we report the somatic PIK3CA alterations in 412 Chinese BC patients, demonstrate the association between the frequency of PIK3CA alterations and the clinicopathological characteristics. We analyze the alteration spectrum within different molecular subgroups, and compare with the data of The Cancer Genome Atlas (TCGA). The ultimate aim is to provide more precise annotations of tumor genomic alterations of Chinese compared to Caucasian in the context of promoting research and application of PI3K inhibitors.

## Materials and methods

### Patients and tumor samples

This study was approved by the ethics committee of Guangdong Provincial People’s Hospital (GDPH) [approval number of 2017312 H(RE)]. The written informed consents were obtained from all participants. A total of 412 Chinese patients of Han nationality who were diagnosed with BC at the GDPH from June 1, 2017 to September 27, 2018 were enrolled in the study. The inclusion criteria are as follows: (1) patients were diagnosed with invasive breast cancer and no chemo or radiotherapy before NGS test; (2) complete clinicopathological information were acquired [sex, age, menstrual status, primary tumor size, axillary lymph node status, pathological type, pathological grade, estrogen receptor (ER), progesterone receptor (PR), and human epidermal growth factor receptor 2 (HER2) status, Ki67 status, distant metastasis status]. Specimens were reviewed by the Department of Pathology at GDPH. The breast cancer molecular subtypes were characterized based on the guideline of St Gallen International Expert Consensus (2019) [13]; and (3) qualified tumor tissue sequencing information.

For The Cancer Genome Atlas (TCGA) cohort, the PIK3CA mutation profiles of Caucasian patients with breast cancer were downloaded from the public website (http://genome-cancer.ucsc.edu) and analyzed. A total of 453 patients were eligible for this study.

### Tissue DNA extraction

DNA from paraffin-embedded breast cancer tissues was extracted using QIAamp DNA FFPE tissue kit (Qiagen, California, US) according to the manufacturer’s instructions. DNA concentration was measured by Qubit dsDNA assay (Life Technologies, California, US).

### NGS library preparation and capture-based targeted DNA sequencing

DNA was subjected to end repair, phosphorylation and adaptor ligation. Fragments of size 200–400 bp were selected by AMPure beads (Agencourt AMPure XP Kit), followed by hybridization with capture probe baits, hybrid selection with magnetic beads and PCR amplification. A high-sensitivity DNA assay was performed to assess the quality and size of the fragments. Indexed samples were sequenced on Nextseq500 sequencer (Illumina, Inc., USA) with pair-end reads.

### Sequence data analysis and identification of variants

Sequence data were mapped to the human genome using BWA aligner 0.7.10. Local alignment optimization, variant calling and annotation were performed using GATK 3.2, MuTect, and VarScan. Variants were filtered using the VarScan fpfilter pipeline, with loci of depth less than 100 filtered out. At least 5 supporting reads were needed for INDELs; while 8 supporting reads were needed for SNVs to be called. According to the ExAC, 1000 Genomes, dbSNP, ESP6500SI-V2 database, variants with population frequency over 0.1% were grouped as SNP and excluded from further analysis.

The reported mutations were further discerned by searching dbSNP (http://www.ncbi.nlm.nih.gov/SNP), ClinVar (http://www.ncbi.nlm.nih.gov/clinvar/), COSMIC(http://cancer.sanger.ac.uk/cosmic), BRCA Exchange (http://www.brcaexchange.org/), and Exome Aggregation Consortium (http://exac.broadinstitute.org/) databases along with PubMed publications to detect novel mutations.

### Statistical analysis

All the computations were performed using the R software (version3.6.0). Pearson’s Chi-square test and Fisher’s exact test were used to study the correlation between the clinicopathological features and the occurrence of PIK3CA mutation. The results with obtained *P* value < 0.05 were considered statistically significant.

## Results

### Association of PIK3CA alterations with clinicopathological features

We examined a total of 412 tumor tissues of Chinese patients with invasive BC. They were divided into PIK3CA altered (*N* = 188) and wild-type (*N* = 224) groups. The clinicopathological features and PIK3CA gene status of these patients are summarized in Table 1. PIK3CA alterations were predominantly found in ER + (49.3%, *p* = 0.024), low ki67 status (58.3%, *p* = 0.007), and low pathological grade tumors (grade I 80%, grade II 53.4% and grade III 35.9%; *p* < 0.001). The PIK3CA alteration status was significantly different among luminal A (57.6%), luminal B (44.6%), Her2-enriched (44.9%) and basal-like molecular subtypes (32.1%). No significant association was found between PIK3CA alterations and onset of age, menopausal status, tumor size, lymph node status, metastasis status, pathological stage, pathological type, PR and Her2 status.

**Table 1:** Clinicopathologic characteristics and PIK3CA status of 412 Chinese patients with invasive breast cancer patients (GDPH cohort)

Characteristics		Wt PIK3CA No.(%) (*N* = 224)	Altered PIK3CA No.(%) (*N* = 188)	*P* value
Age (years)	< = 50	132 (56.2)	103 (43.8)	0.456
	> 50	92 (52)	85 (48)	
Sex	Male	1(100)	0	–
	Female	223(54.3)	188(45.7)	
Menopausal status	Pre	129 (56.1)	101 (43.9)	0.46
	Post	94 (51.9)	87 (48.1)	
	Missing	1	0	
Pathologic T	T1	80 (52.3)	73 (47.7)	0.303
	T2	115 (53.5)	100 (46.5)	
	T3	20 (71.4)	8 (28.6)	
	T4	9 (56.2)	7 (43.8)	
Pathologic N	N0	105 (52.2)	96 (47.8)	0.412
	N1	56 (51.9)	52 (48.1)	
	N2	44 (59.5)	30 (40.5)	
	N3	19 (65.5)	10 (34.5)	
Pathologic M	M0	207 (53.9)	177 (46.1)	0.476
	M1	17 (63)	10 (37)	
	Missing	0	1	
Pathologic stage	IA	49 (48)	53 (52)	0.468
	IIA	71 (59.2)	49 (40.8)	
	IIB	36 (50.7)	35 (49.3)	
	IIIA	31 (53.4)	27 (46.6)	
	IIIB	3 (42.9)	4 (57.1)	
	IIIC	17 (65.4)	9 (34.6)	
	IV	17 (63)	10 (37)	
	Missing	0	1	
Pathological grade	I	3 (20)	12 (80)	** < 0.001**
	II	89 (46.6)	102 (53.4)	
	III	127 (64.1)	71 (35.9)	
	Missing	5	3	
Pathological type	Ductal	193 (53.5)	168 (46.5)	0.184
	Lobular	4 (30.8)	9 (69.2)	
	Others	27 (71.1)	11 (28.9)	
ER status	Positive	149 (50.7)	145 (49.3)	**0.024**
	Negative	75 (63.6)	43 (36.4)	
PR status	Positive	143 (52.4)	130 (47.6)	0.303
	Negative	81 (58.3)	58 (41.7)	
HER2 status	Positive	60 (51.3)	57 (48.7)	0.306
	Negative	157 (57.5)	116 (42.5)	
	Missing	7	15	
Ki67 status	< 14	40 (41.7)	56 (58.3)	**0.007**
	> = 14	182 (58)	132 (42)	
	Missing	2	0	
Molecular subtype	Luminal A	36 (42.4)	49 (57.6)	**0.03**
	Luminal B	124 (55.4)	100 (44.6)	
	HER2-enriched	27 (55.1)	22 (44.9)	
	Basal-like	36 (67.9)	17 (32.1)	
	Missing	1	0	
ER/HER2 subtype	ER + /HER2-	118(51.3)	112(48.7)	0.05
	ER + /HER2 +	31(48.4)	33(51.6)	
	ER-/HER2 +	33(56.9)	25(43.1)	
	ER-/HER2-	42(70.0)	18(30.0)	

*GDPH* Guangdong Provincial People’s Hospital, *No.* number, *Wt* wild type, *PIK3CA* phosphatidylinositol-4,5-bisphosphate 3-kinase catalytic subunit alpha, *ER* estrogen receptor, *PR* progesterone receptor, *HER2* human epidermal growth factor receptor 2

### The characteristics of PIK3CA alterations in GDPH and TCGA-white cohort

**Fig. 1:**
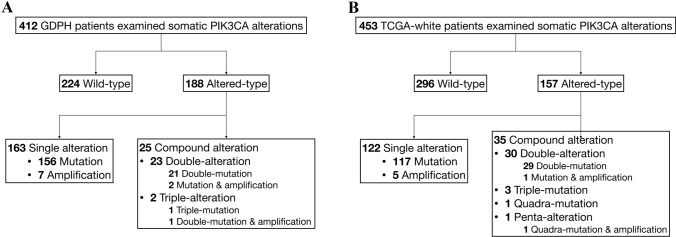
PIK3CA somatic alteration distribution across breast cancer population of GDPH and TCGA-white cohorts. **a** Distribution in GDPH population. **b** Distribution in TCGA-white population. *PIK3CA* phosphatidylinositol-4,5-bisphosphate 3-kinase catalytic subunit alpha, *GDPH* Guangdong Provincial People's Hospital, *TCGA* The Cancer Genome Atlas

In the GDPH cohort, 49 somatic alterations were detected in 188 out of 412 patients, with a gross alteration rate of 45.6% (Fig. 1a). 163 patients had single alterations (mutation or amplification) and 25 had compound alterations. In the TCGA-white cohort, 43 somatic alterations were detected in 157 out of 453 patients (alteration rate of 34.7%) (Fig. 1b), which is lower than that of the GDPH cohort (*p* < 0.001). 127 patients had single alterations and 30 harbored compound mutations.

**Fig. 2:**
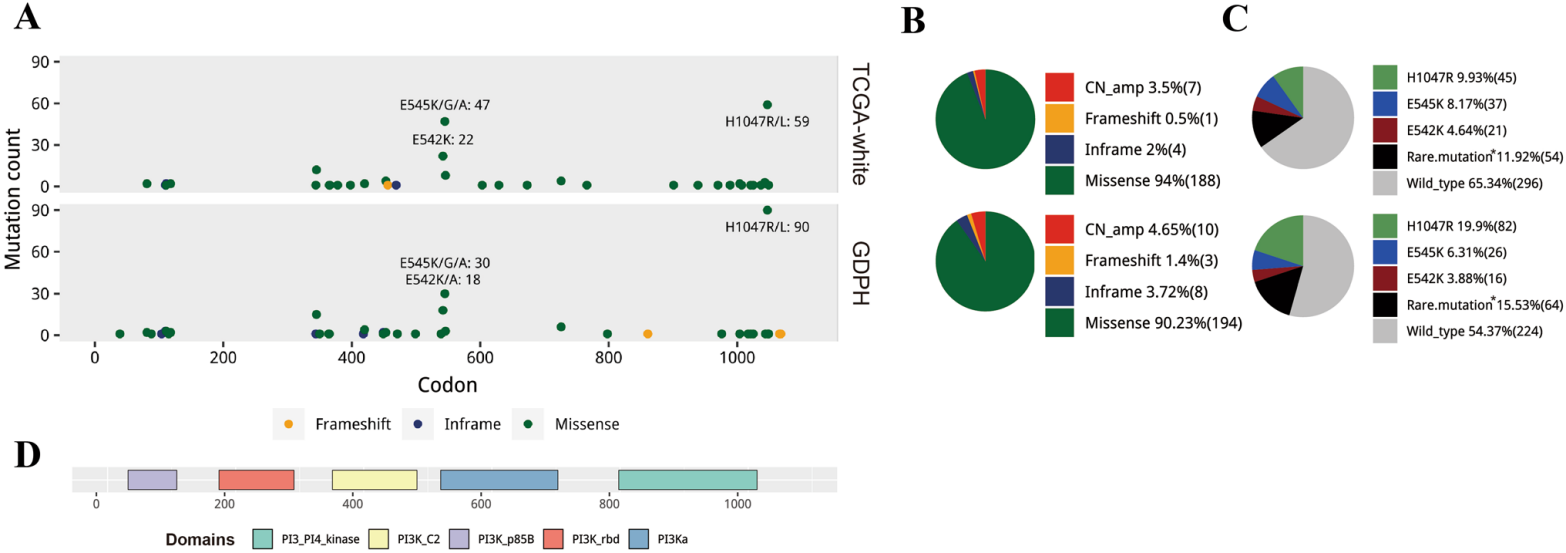
PIK3CA alteration spectrum in breast cancer of the TCGA and GDPH cohorts. **a** Distribution of mutation across the gene. **b** Pie charts showing the fraction of alteration types. **c** Pie charts showing the fraction of mutation sites. **d** Conserved and functional domains of PIK3CA gene. *Rare mutation: defined as PIK3CA mutations except three common mutation sites: p.E545K, p.E542K and p.H1047R. *PIK3CA* phosphatidylinositol-4,5-bisphosphate 3-kinase catalytic subunit alpha, *GDPH* Guangdong Provincial People's Hospital, *TCGA* The Cancer Genome Atlas, *CN_amp* copy number amplification

The PIK3CA alteration type and mutation site were studied and illustrated in Fig. 2. The predominant alteration type was missense mutation (GDPH 90.23%, TCGA-white 94%). Both cohorts had similar PIK3CA mutation site. The PIK3CA mutations occurred in several exons (exon1, 2, 4, 5, 7–9, 13, 15, 17, 19, 20), with the most frequent location in exon 9 and 20 for both GDPH (73.7%) and TCGA-white population (66.0%). The PIK3CA mutation sites with mutation rate ≥ 1% in GDPH cohort were showed in Table 2. The fraction of three common mutation sites including p.E545K, p.E542K and p.H1047R were showed in Fig. 2c. The TCGA-white cohort had almost similar common mutations rate among all altered PIK3CA compared to the GDPH cohort (65.6% vs 66.0%, *p* = 0.95). However, p.H1047R mutation occupied a significantly higher proportion in GDPH cohort than that of TCGA-white cohort, in all cases (19.9% vs 9.9%, *p* < 0.001) or in common mutations (66.1% vs 43.7%, *p* = 0.01). In addition, 9 novel mutation sites of PIK3CA were detected in the GDPH cohort (Table 3).

**Table 2:** PIK3CA mutation sites with mutation rate ≥ 1% in GDPH cohort and corresponding rate in TCGA-white cohort

Exon	Nucleotide change	Mutation site	GDPH No. (%) (*N* = 412)	TCGA-white No. (%) (*N* = 453)	*P* value
20	c.3140 A > G	p.H1047R	82(19.9)	45(9.9)	** < 0.001**
9	c.1633 G > A	p.E545K	27(6.6)	37(8.2)	0.435
9	c.1624 G > A	p.E542K	17(4.1)	21(4.6)	0.743
4	c.1035 T > A	p.N345K	14(3.4)	8(1.8)	0.137
20	c.3140 A > T	p.H1047L	8(1.9)	8(1.8)	1.000
13	c.2176G > A	p.E726K	6(1.5)	4(0.9)	–
7	c.1258 T > C	p.C420R	4(1)	1(0.2)	–

*PIK3CA* phosphatidylinositol-4,5-bisphosphate 3-kinase catalytic subunit alpha, *GDPH* Guangdong Provincial People's Hospital, *TCGA* The Cancer Genome Atlas, *p.* protein sequence, *No.* number

**Table 3:** Novel mutation sites of PIK3CA detected in GDPH cohort

Mutation site	Codon	Mutation type	Exon
p.V344_V346dup	344	Inframe	4
p.E418_L422 > V	418	Inframe	7
p.P449_E453 > Q	449	Inframe	7
p.P449_V461del	449	Inframe	7
p.P449S	449	Missense	7
p.E798N	798	Missense	15
p.Q861fs	861	Frameshift	17
p.E976K	976	Missense	19
p.A1066fs	1066	Frameshift	20

*PIK3CA* phosphatidylinositol-4,5-bisphosphate 3-kinase catalytic subunit alpha, *GDPH* Guangdong Provincial People's Hospital, *TCGA* The Cancer Genome Atlas, *p.* protein sequence, *No.* number

### Spectrum of PIK3CA alterations across ER/HER2 subtypes

**Fig. 3:**
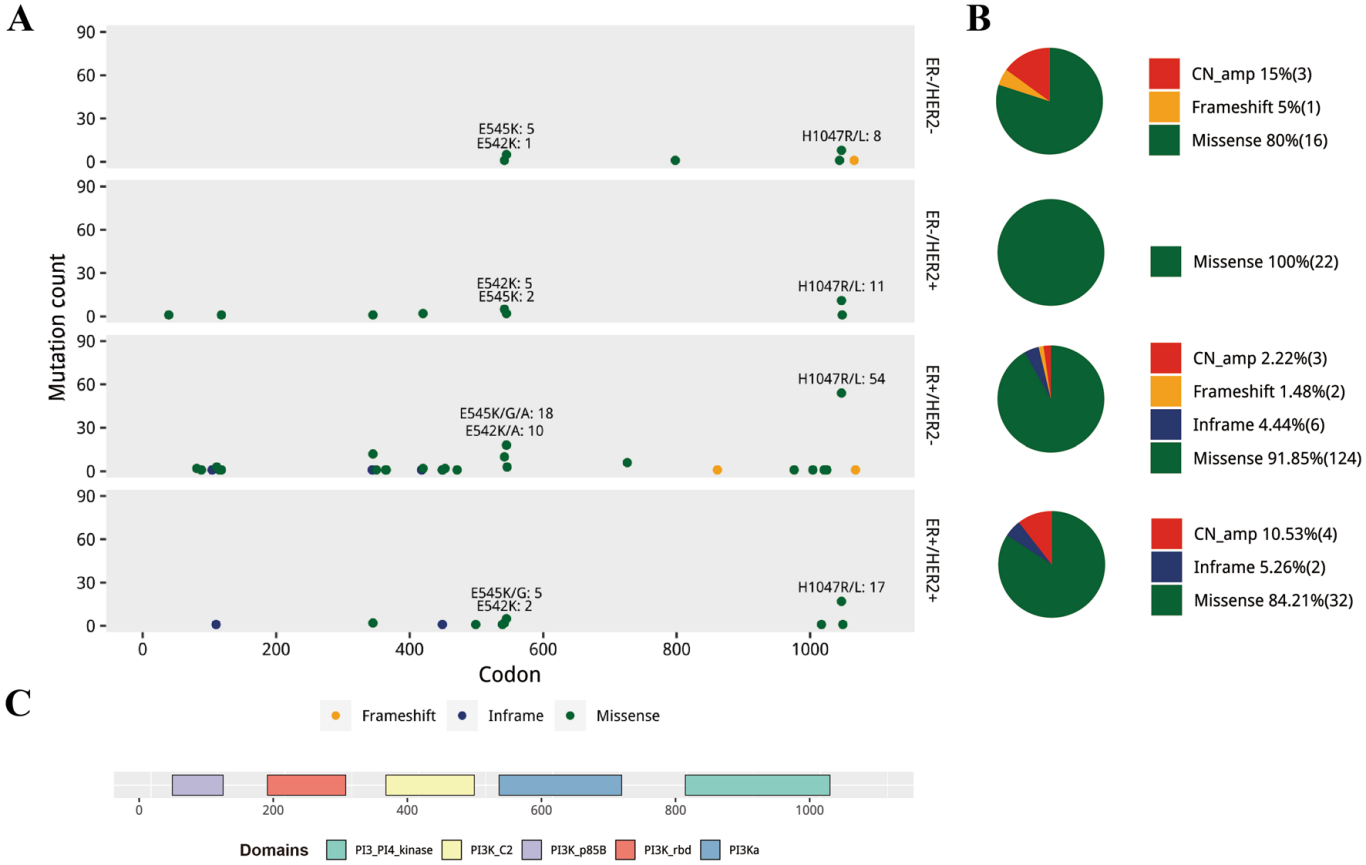
PIK3CA alteration spectrum across four ER/HER2 subgroups in breast cancer of GDPH cohort. A. Distribution of mutation across the gene. B. Pie charts showing the fraction of alteration types. C. Conserved and functional domains of PIK3CA gene. *PIK3CA* phosphatidylinositol-4,5-bisphosphate 3-kinase catalytic subunit alpha, *GDPH* Guangdong Provincial People's Hospital, *TCGA* The Cancer Genome Atlas, *CN_amp* copy number amplification

The PIK3CA alteration spectrum was studied in detail by dividing the GDPH specimens into four ER/HER2 subtypes (Fig. 3). PIK3CA alterations occurred at the highest frequency in ER + /HER2 + (51.6%) tumors, followed by the ER + /HER2-(48.7%) and ER-/HER2 + (43.1%) tumors, and the lowest in ER-/HER2-(30.0%) tumors (Table 1). Missense mutation was the primary alteration type for four subtypes, while copy number amplification occupied considerable proportion in ER-/HER2- type (19.05%). Common mutations distributed extremely alike across subtypes with rates among all altered PIK3CA as 66.1% in ER + /HER2 −, 66.7% in ER + /HER + , 64.0% in ER-/HER2 + and 66.7% in ER −/HER2 − subtype. Because of the limited sample size, none of the three common mutations were separately analyzed.

## Discussion

In the current study, we explored the frequency and spectra of somatic altered PIK3CA gene by high-resolution genomic sequencing in a large cohort of 412 Chinese patients with invasive BC. To the best of our knowledge, this is one of the largest studies to describe the alteration spectrum of PIK3CA gene in Chinese BC patients of Han nationality compared with Caucasian. The frequency of PIK3CA alteration is 45.6% in our study, which is slightly higher than that of TCGA-white data (34.7%) and other literature data (7–36%) in Chinese [14–16], 14–45% in other Asians [11, 17, 18], 18–40% in Americans [19] and 13–45% in Europeans [20, 21]. The disparity of PIK3CA gene mutation rate in different studies could be explained by ethnicity, sample size, inclusion criteria or gene sequencing methods.

In accordance with previous studies, the majority of PIK3CA mutations occurred in three hotspot sites, namely E542K and E545K in helical domain and H1047R in kinase domain [11]. Interestingly, there is a significantly larger proportion of p.H1047R mutation in our study than the TCGA Caucasian cohort. The H1047R mutation enhances the interaction of the p110 kinase domain with cell membranes, while the E542K and E545K mutations disrupt the inhibitory interface with p85 [22]. Due to different functional mechanisms, 3 hotspot sites may cause different damage through downstream signaling. An in vitro study showed that the helical domain mutants produced a more aggressive phenotype than kinase domain mutants [23]. However, one study suggested that the mere existence of mutant E545K may not be as harmful as H1047R [24]. As for utilization of antineoplastic drugs, limited studies have focused on the relationship between specific PIK3CA alteration site and effectiveness of drugs [25]. In a retrospective analysis, Binghe Xu, etc. suggested that PIK3CA/H1047R mutations may be a potential biomarker of sensitivity to everolimus, wherein H1047R mutated patients had longer PFS than wild-type or other mutant forms of PIK3CA. Analyses in the neoadjuvant setting suggested that mutations in exon 9 conferred a higher sensitivity to pictilisib when compared with mutations in exon 20 [26]. In general, two puzzles remain to be clarified about clinical significance of PIK3CA alteration sites: (1) extent of harmfulness for different PIK3CA alteration domains; and (2) PIK3CA alteration site as a biomarker for patients who benefit most from PI3K inhibitors.

Another question is the relationship between PIK3CA alterations and clinicopathological characteristics. In present study, PIK3CA alterations happen more in BC with low pathological grade, ER positive and low Ki67 index. The positive correlation between PIK3CA alteration and better prognosis has also been previously reported [20, 27, 28]. A large-scale systematic review of BC clinical studies involved 2587 cases showed that patients with tumors harboring a PIK3CA mutation have a better clinical outcome, especially for postmenopausal women with ER + BC [29]. However, recent molecular profiling data from MBC patients seem to indicate that in advanced HR + /HER2 − BC, a PIK3CA mutation would lead to resistance to chemotherapy and a poor outcome [30]. On all accounts, improved understanding of the clinical impact of PIK3CA alterations is critical to prevent or explain therapeutic failures and develop optimal personalized therapeutics against breast cancer.

Additionally, the relevance between PIK3CA alteration spectrum and ER/HER2 molecular subtypes has also been detected in our study. PIK3CA alterations were more prevalent in ER + tumors and the least in the ER−/HER2−(30.0%) subtype, in agreement with previous studies [5, 21, 31]. Despite the overall low prevalence of PIK3CA mutations in TNBC, the proportion is not negligible. The finding of Jiang etc. [4] stated that Chinese TNBC cases demonstrate 18% PIK3CA mutations, we found the mutation rate of 30%. Therefore, high PIK3CA mutation may be a remarkable feature of Chinese TNBC patients. Meanwhile, ER−/HER2− subtype in our study is enriched for frameshift and DNA copy number amplification, which was similarly observed previously [32], suggesting the genomic instability in TNBC subtypes. Several clinical studies are trying to determine the potential benefit of PIK3CA inhibitors in different BC subtypes. Although the efficacy of additional PIK3CA inhibitor shows lack of significance in the early setting [33, 34], the combined use with existing therapies in heavily pretreated MBC patients demonstrates substantial prospective [10, 35]. On all accounts, a biomarker-driven approach to identify optimal clinical choices in the background of PIK3CA gene alterations is warranted.

In conclusion, our study showed that PIK3CA alteration is a common event in Chinese BC patients and has a unique spectrum compared to that of Caucasians. The distribution patterns of PIK3CA alteration are distinct across four molecular subtypes based on ER/HER status. Future clinical trials should assess PIK3CA alteration characteristics as biomarkers to predict therapeutic response to PI3K inhibitors.
